# Unexpected increase in structural integrity caused by thermally induced dwarfism in large benthic foraminifera

**DOI:** 10.1098/rsos.231280

**Published:** 2024-04-10

**Authors:** Danna Titelboim, Nikita J. Rothwell, Oliver T. Lord, Robert L. Harniman, Leanne A. Melbourne, Daniela N. Schmidt

**Affiliations:** ^1^ School of Earth Sciences, University of Bristol, Bristol, UK; ^2^ School of Chemistry, University of Bristol, Bristol, UK; ^3^ Earth and Planetary Sciences Department, American Museum of Natural History, New York, NY, USA

**Keywords:** ocean warming, thermal stress, calcification, structural integrity, morphological adaptation

## Abstract

Climate change is predicted to negatively impact calcification and change the structural integrity of biogenic carbonates, influencing their protective function. We assess the impacts of warming on the morphology and crystallography of *Amphistegina lobifera*, an abundant benthic foraminifera species in shallow environments. Specimens from a thermally disturbed field area, mimicking future warming, are about 50% smaller compared with a control location. Differences in the position of the ν1 Raman mode of shells between the sites, which serves as a proxy for Mg content and calcification temperature, indicate that calcification is negatively impacted when temperatures are below the thermal range facilitating calcification. To test the impact of thermal stress on the Young's modulus of calcite which contributes to structural integrity, we quantify elasticity changes in large benthic foraminifera by applying atomic force microscopy to a different genus, *Operculina ammonoides*, cultured under optimal and high temperatures. Building on these observations of size and the sensitivity analysis for temperature-induced change in elasticity, we used finite element analysis to show that structural integrity is increased with reduced size and is largely insensitive to calcite elasticity. Our results indicate that warming-induced dwarfism creates shells that are more resistant to fracture because they are smaller.

## 1. Introduction

Changes in the physical and chemical characteristics of the ocean due to climate change affect ecosystems in all regions, including shallow and coastal organisms [1]. As these environmental changes intensify, an adverse effect is predicted on the growth, reproduction, recruitment and other physiological functions of most marine organisms. Marine calcifiers play an important role as marine ecosystem engineers, as they produce carbonate shells and skeletons that protect the organisms from predation and support ecosystem functions including habitat formation, reducing storm impacts and helping to reduce erosion of coastlines.

Previous studies demonstrated the effects of climate change on the morphology and chemical composition of carbonate skeletons [2–5], which influence their mechanical properties. For example, changes to the cellular structure within coralline algae can change internal stress distributions [6], while increased incorporation of Mg into the crystal lattice increases the solubility and hardness of the carbonate material as demonstrated in sea urchin teeth and corallines [7,8]. A few studies suggest that these changes create weaker structures in bivalves and coralline algae [9–11]. However, as these studies are limited in taxonomic diversity and the range of conditions studied, it precludes assessment of the impact of warming on the ability of marine calcifiers to provide their ecosystem services.

Large benthic foraminifera (LBF) are prominent carbonate producers in shallow water environments and contribute to the cementation of structures such as reefs. They also supply huge amounts of sand that maintain the coastlines of low-lying islands [12,13]. Their calcite shells fulfil a crucial role in the protection of the organism from external stressors such as predation and wave action. The level of protection is determined by the structural integrity of the shell, which is largely dictated by its morphology and by the material properties of the calcite [14]. Plasticity in response to variability in environmental conditions or related to adaption is often expressed morphologically in foraminifers [15]. For example, in symbiont-bearing benthic foraminifera, low light levels may cause calcification of thinner shell walls that allow better penetration of light to their symbionts [16], while thermal stress results in smaller and thinner shells [4,17–19]. In contrast to calcification and carbonate productivity, very little is known about the impacts of warming on the material properties that control the strength of the shell and its ability to protect the organism. It is well established, however, that warming increases the Mg content of biogenic and non-biogenic carbonates, subtly altering their crystal structure and in turn affecting solubility, hardness and elasticity [8,20–22]. While specific effects on foraminifera have not been quantified to date, we hypothesize that future climate change will, via changes in morphology and material properties, influence the structural integrity of their shells and thus alter their ability to protect the organism and act as ecosystem engineers.

An effective approach to examine the structural integrity of shells is finite element analysis (FEA). FEA is a computational technique used to approximate physical behaviour under varying experimental conditions, predicting stress and strain by subdividing a large system into smaller simpler parts (i.e. finite elements). This technique was originally developed for resolving mathematical and engineering questions but has become increasingly common in palaeontology [23]. More recently, it has been applied to quantifying the effect of environmental change on the structural integrity of calcifying organisms [9,11,24,25] including branching and massive corals [26] and coralline algal skeletons [6,10]. These examples suggest a higher risk of fracture as storm surges become more intense in the future [27]. Despite their environmental significance, there is only one study that uses FEA to understand the structural impacts of morphological changes in LBF shells, focusing on their extraordinarily large size and complex internal structures for unicellular organisms but not on changes in structural integrity resulting from environmental changes [28].

**Figure 1.**
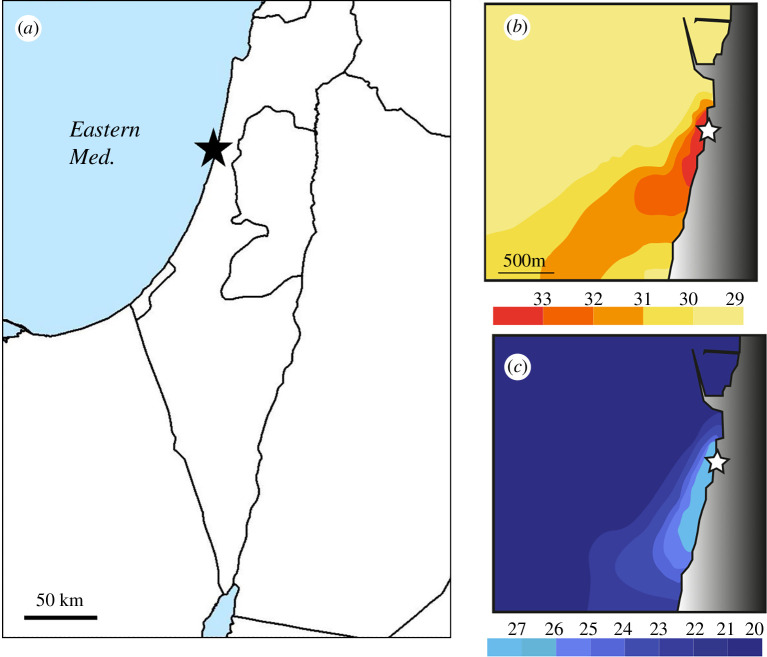
Location map (*a*) and illustration of the thermally disturbed area in spring (*b*) and autumn (*c*). Star represents location of warm water discharge.

In this study, we use FEA to determine how changes in both calcification and material properties resulting from warming will affect the structural integrity of LBF shells. We use samples collected from a thermally disturbed environment created by an electrical power plant discharging warm water into the Eastern Mediterranean (figure 1). Using this site takes advantage of the ecosystem being exposed to warmer temperatures but otherwise to normal natural conditions including seasonal variability. Thus, the specimens have adapted to the increased warmth over decades and include any genotypic and ecophysiological changes that have accrued over many generations. We quantify changes in growth and calcite crystal structure under elevated temperatures in this field laboratory and a control station to investigate their effect on material properties and structural integrity. We focus on *Amphistegina lobifera* which is very common and contributes to carbonate production in many shallow-water environments [12,29,30]. The taxon has a well-defined temperature and salinity range across its wide geographical distribution [31] with geographical range expansion driven by warming as has been exemplified by their invasion of the Eastern Mediterranean from the Red Sea [32–34]. Their thermal optimum for calcification is 20–25°C with a strong reduction in calcification at 30–32°C and complete inhibition at 35°C [35,36]. This results in exclusion from areas with high temperatures such as anthropogenically warmed areas of the Eastern Mediterranean and an inability to tolerate future warming [37].

## 2. Material and methods

### 2.1. Study area

Warm water discharged by a local power plant along the northern Mediterranean coast of Israel locally enhances temperatures, creating a thermal gradient that follows the seasonal temperature cycle (figure 1). We collected shells of *A. lobifera* from a warm station at the southern edge of the thermally disturbed area (Givat Olga; 32.447° N, 34.8780° W) and a control area (Nachsholim; 32.6231° N, 34.9195° W). The shells were collected under permission number 2013/39805 issued by the Israel Nature and National Park Authority. The sites were monitored monthly for their foraminiferal populations and environmental conditions from April 2013 to March 2014 so as to include the full seasonal cycle [37]. The temperatures in each station were recorded every 15 min using data loggers that indicate the warm station is 0.4–1.9°C warmer compared with the control station (monthly averaged). Monitoring of other environmental parameters (salinity, pH, nutrient concentrations, dissolved oxygen and water chemistry) were recorded monthly upon sample collection and were similar between both locations ([37] and electronic supplementary material, table S1). During the sampling period, daily averaged maximum temperatures were recorded in August at 30.8°C in the control station and 32.1°C in the warm station, and minimum temperatures were reached in December with 12.7°C and 13.6°C at the control and warm stations, respectively [37]. The warmer station represents the edge of *A. lobifera*’s distribution within the thermally disturbed area, about 2.3 km from the warm water discharge (figure 1) suggesting that these temperatures are close to the taxon’s limit and that the specimens must be under thermal stress. Note that *A. lobifera* was completely absent at this warm station between April and July when temperatures were between 26°C and 33°C [37].

### 2.2. Computed tomography scans

Morphological differences in *A. lobifera* between the control and warm station have been quantified using computed tomography (CT). Six to eight specimens from each station per month, totalling 45 specimens from the warm and 64 from the control station were collected roughly every other month, have been scanned to cover the annual cycle at each station. The samples were scanned using a Nikon XTH 225 ST CT scanner with beam conditions of 80 kV and 88 μA and an exposure time of 0.708 ms. Each scan consists of approximately 380 images, with an average voxel size of 3.2 μm. Images have been reconstructed and processed using the three-dimensional imaging software AVIZO (v. 2020.2, Mercury Computer Systems Ltd, Chelmsford, MA, USA, www.tgs.com). Each specimen was individually segmented to produce a three-dimensional rendering of the shell. Segmentation was done manually on each of the specimens to detect the outline of the shells. Any infilling within the shells was removed by assigning a grey-scale value to each pixel representing the different X-ray absorption properties of calcite versus other material in the sample (following Schmidt *et al*. [38]). After segmentation, the overall volume, calcite volume, surface area, diameter and calcite-to-volume ratio (which we use as an indicator of shell thickness) were quantified. Shell size was measured using multiple parameters (overall shell volume, calcite volume, shell surface area and shell diameter) that positively correlated with each other, thus for convenience, size will be represented by overall shell volume. The other measures of size can be found in electronic supplementary material, table S2. Differences between specimens collected from each site and during different months were estimated using the Kruskal–Wallis test.

### 2.3. Raman spectroscopy

To test the impact of warming on the crystal structure of *A. lobifera* shells, we performed Raman spectroscopy on the field specimens. We examined the internal symmetric stretching mode of the CO_3_^2−^ anion (ν1, approximately 1088 cm^–1^) because it has the highest amplitude of all calcite Raman modes, hence it provides the most accurate results. This measurement was used as a proxy for Mg incorporation into the calcite lattice [39], since progressive substitution of the larger Ca cation by the smaller Mg cation reduces the average C–O bond length, increasing its vibrational frequency and shifting the Raman mode to higher wavenumbers (cm^−1^).

We measure changes in position of the ν1 mode in *A. lobifera* collected from the control and warm station to compare shells that calcified under different thermal conditions. Aiming to cover both the warmest and coldest temperatures, we measured Raman spectra of shells collected from the control station in August (warmest month, 30°C), October (25°C) and February (coldest month, 17°C), and from the warm station in August (warmest month, 31°C). For each of these, we measured two spots on the outermost part of the shell (i.e. the last calcite produced) of five specimens to include both intra- and inter-specimen variability, resulting in a total of 40 measurements. The measurement of the outermost chamber of specimens collected at the warmest and coldest months captures the calcification events just before calcification stopped due to any thermal thresholds reached (both high and low). Differences between specimens from each thermal condition were estimated using the Kruskal–Wallis test and a post hoc pairwise comparison Dunn test.

Raman spectra were collected on a confocal Jobin Yvon Horiba T64000 instrument equipped with a 1200 lines cm^–1^ diffraction grating (yielding a spectral resolution of approximately 1 cm^–1^), a Laser Quantum Torus 532 nm solid-state laser source operating at 100% power (approximately 500 mW) and a 100× objective, yielding a diffraction-limited focal volume with a diameter of approximately 4 μm. Each spectrum was produced from 10 accumulations, each with an exposure time of 10 s. Building on [4], two calibration lamps (Hg and Kr) were lit throughout the analytical session, such that each spectrum contained three emission lines of precisely known frequency. The wavenumber–pixel relationship of the detector was calibrated using a weighted linear orthogonal distance regression to the difference between the measured and known frequencies of the calibration peaks, so that all reported peak positions are corrected for both instrument offset and temperature fluctuations during the analytical session (i.e. instrument drift). In this way, every spectrum contains its own internal calibration, eliminating the effects of instrumental offset and drift during the analytical session. The centre positions of the ν1 Raman modes were determined by fitting to Pseudo-Voigt functions in the software package fityk [40]. Repeatability based on multiple analyses of the Hg and Kr calibration lamps is estimated as 0.2 cm^–1^.

### 2.4. Young’s modulus

To determine Young’s modulus, for use in subsequent FEA analysis, we performed atomic force microscopy (AFM). Often in FEA, a standard Young’s modulus for calcite will be applied for all experiments. Here, though, we wanted to perform a sensitivity analysis to quantify how much Young’s modulus could change in response to environmentally driven changes in material properties such as the Mg content. Ideally, AFM analysis would have been performed on the specimens we collected from the field site. However, as individuals have not necessarily calcified new chambers at the time of field collection [41], we would not be able to link our measurements with calcification temperatures or levels of stress.

Thus, we decided to explore specimens cultured under laboratory conditions to determine the impact of calcification temperature on material properties. We choose to culture the genus *Operculina ammonoides* as it has the same hyaline perforated shell type as *A. lobifera* produced under the same underlying biomineralization mechanism [42] but does not have the same low physiological thermal threshold as *A. lobifera,* which would have made it challenging to obtain enough new calcite for measurement at temperatures above 30°C.

#### 2.4.1. Laboratory culturing experiment

Twenty *O. ammonoides* specimens were cultured at 25°C and 35°C, representing normal and thermally stressed conditions. Calcification of new chambers was identified by Calcein staining (following Titelboim *et al*. [41]). We compare differences between chambers that grew before the culturing phase under field conditions with those that precipitated in culture under both temperature treatments (25°C and 35°C).

#### 2.4.2. Atomic force microscopy analysis

AFM was conducted at ambient conditions on sections of polished samples embedded in resin using a Multi-Mode VIII microscope with Nanoscope V controller (Bruker, CA, USA). Topographic maps of the crystal structure in chamber walls were collected using a Fast-scan head unit and PeakForce feedback control with SCANASYST-AIR-HR cantilevers with a nominal spring constant and tip radius of 0.4 N m^–1^ and 2 nm, respectively. Force curves were collected on chamber walls using a hand-crafted MDNISP-HS probe consisting of a stainless-steel cantilever with ground single-crystal diamond tip. The MDNISP-HS probe has a spring constant of 412.31 N m^–1^ and a tip radius of 40 nm. Deflection sensitivity was calibrated on a fused-silica standard. Sets of 20 force curves were collected in seven different locations on each chamber wall, for a minimum of 140 measurements per chamber. Young’s moduli and associated error were then calculated by fitting a Derjaguib–Muller–Toporov (DMT) model to 70% of each unloading curve and taking the average across all measurements for each chamber wall. This was repeated on three shells that calcified new chambers at 25°C and another three that precipitated at 35°C. In the specimens that calcified at 25°C, the last and penultimate chambers were measured. In contrast, in the specimens that calcified at 35°C, only the last chamber was measured as it is the only calcite created at 35°C due to the high stress level at this treatment. In each case, the Young’s modulus of a ‘natural chamber’, created before collection from the field, was also measured as a control value for the sample. The measurement from each treatment yields uncertainties in the mean that do not overlap. Thus, the two populations have statistically significant differences in their Young’s moduli.

### 2.5. Finite element analysis

**Figure 2.**
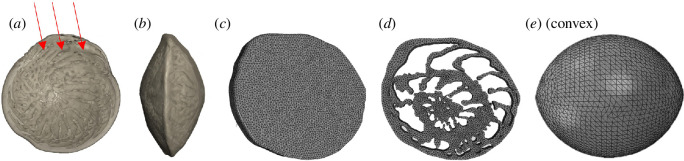
Image of the examined species *A. lobifera* rendered from a CT scan in spiral (*a*) and side (*b*) views, and models used for FEA representing the external (*c*) and internal (*d*) structures, and overall convex shape (*e*). Arrows represent force direction applied on the models for FEA.

To estimate the effect of changes in morphology and Young’s modulus upon structural integrity, we used FEA to produce stress distribution models. Using the commercial software package Abaqus CAE 2020, we created three simplified models representing the two-dimensional external shape and internal morphology, based on CT scans (figure 2*c,d*), and three-dimensional external model with a biconvex oval shape representing a more realistic and general shell structure (figure 2*e*). To represent characteristics for both future and current warming scenarios, we used the average diameter and volume of the CT scans from the warm and control stations (table 1), which effectively downsized the warm station models by a factor of 0.56 compared with the control station models (as described in the §3). The models were meshed to minimize the influence of element size: for thetwo-dimensional models element sizes of 0.2 and 0.02 were used for the control and the warm station models, respectively, and an element size of 2 was used for the biconvex three-dimensional models. All specimens were assumed to have a homogeneous composition of isotropic material with a linear elasticity, a Poisson’s ratio of 0.32 [43] and a Young’s modulus of 45.7 and 84.0 GPa for the control and ‘warm’ models, respectively (as measured using AFM described above).

**Table 1.** Summary of the CT size measurements for FEA.

month	site	mean shell diameter (mm)	mean shell volume (mm^3^)	mean temperature
Apr 2013	control	0.87	0.19	20.3
May 2013	control	1.76	0.69	23.8
Aug 2013	control	0.92	0.12	30.4
Oct 2013	control	1.36	0.24	24.9
Dec 2013	control	1.23	0.41	17.9
Jan 2014	control	1.90	0.56	17.2
Feb 2014	control	1.21	0.34	17.5
Aug 2013	warm	0.73	0.08	30.8
Oct 2013	warm	0.85	0.11	26.8
Dec 2012	warm	1.13	0.24	19.2
Jan 2014	warm	1.15	0.26	18
Feb 2014	warm	1.00	0.18	18.3
Mar 2014	warm	1.24	0.36	19.7

Drag forces have been used to represent the impact of wave loading, as a compressional force. We applied the force to the side of the specimen that would be exposed to water flow, while the opposite side was constrained to represent the attachment of the foraminifera to the surface of an algae or substrate (see figure 2). A force of 20 kPa was used, based on drag experienced by algae at comparable depths of approximately 20 m [6]. The analyses were repeated using a loading value of 30 kPa to test the stress distribution under increased force conditions, that is, future increased wave action. Finally, we tested whether morphology or material properties had the greater effect on average stress values. Only the three-dimensional biconvex model showed changes in stress distribution. Using that model, we proceed with two tests: varying size at constant elasticity and varying elasticity at constant size while keeping element size, boundary and loading conditions (20 kPa) constant in both. For each output, average von Mises stresses were recorded as a predictor of failure in brittle materials [6]. The differences between these model outputs were estimated based on uncertainties in the mean that do not overlap, indicating significant differences in their von Mises stress values. The strain and stress were calculated from the FEA models using the mathematical equations detailed in Mathematics of FEA [23].

### 2.6. Statistical analysis

Abundance data of *A. lobifera* in both stations was statistically analysed based on data published in [37], Shapiro–Wilk test yielded *p*‐value < 0.01, indicating non-normal distribution. For the CT and Raman data, the Shapiro–Wilk test yielded *p*-values of 0.08 and 0.04, respectively. However, in both cases, the quantile–quantile plots showed deviations from normality, particularly in the tails, suggesting a non-normal distribution. Therefore, we analysed all three datasets using the non-parametric Kruskal–Wallis test, and to compare the ν1 Raman mode position between all groups, we conducted post hoc pairwise comparisons using the Dunn test. For the AFM and FEA, as the uncertainties in the mean were highly distinct with no overlap, we did not apply any statistical test as the significant differences were clear.

## 3. Results

### 3.1. Morphology, crystallography and material properties

**Figure 3.**
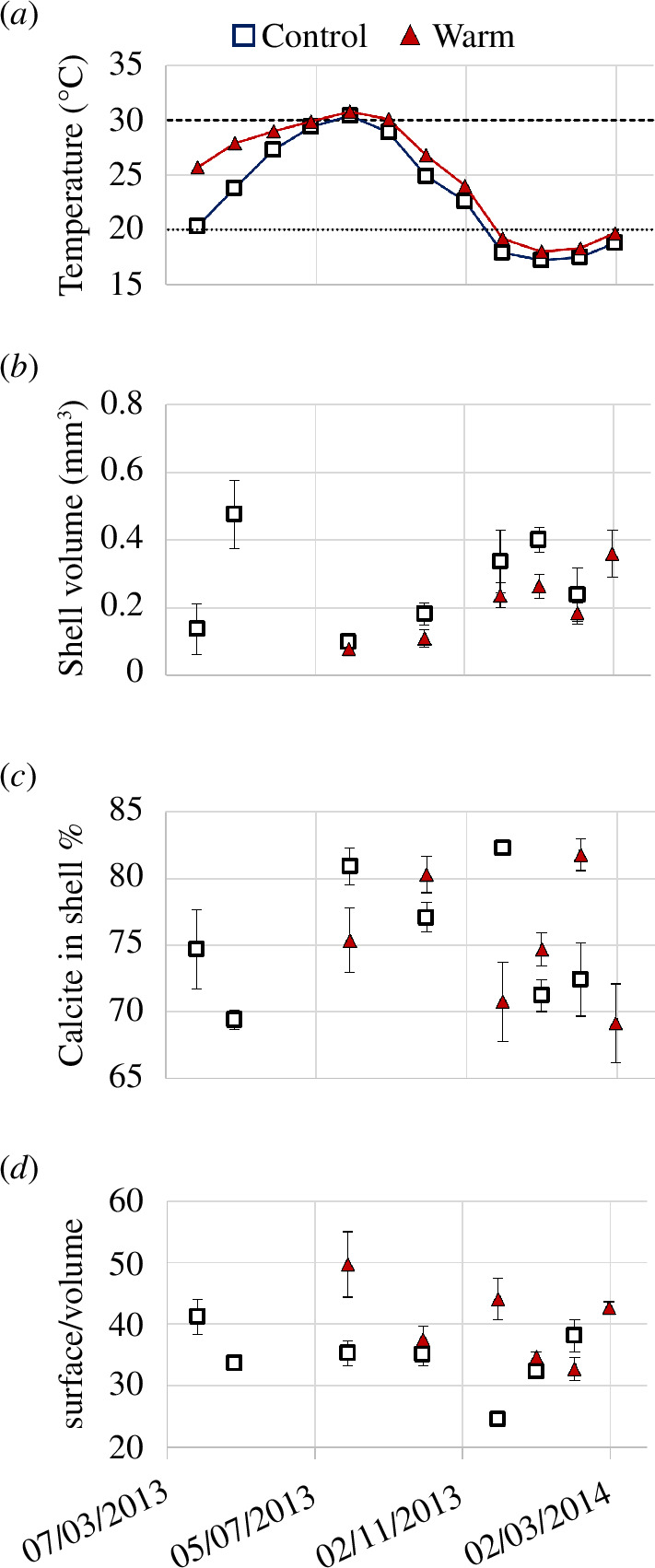
Monthly averaged temperatures (*a*) shell volume (*b*) per cent calcite shell (i.e. shell thickness) (*c*) and surface-to-volume ratios (*d*) at the control (empty squares) and warm (red full triangles) stations. *Amphistegina lobifera* were absent up to August at the warm station resulting in data gaps. Error bars represent the standard error in the mean.

The response of *A. lobifera* to elevated temperature is indicated by the 90% reduction in abundance in the warm compared with the control station (Kruskal–Wallis *χ* ^2^ = 30.774, d.f. = 1, *p*‐value < 0.01), and the operation of a different seasonal cycle, including the absence of shells between April and July in the warm station [37]. The change in the yearly reproductive cycle results in larger shells in the control compared with the warm station consistently throughout the year (figure 3). The overall shell volume at the control station is significantly higher than that of the warm station (Kruskal–Wallis *χ* ^2^ = 9.2438, d.f. = 1, *p*‐value < 0.01) creating shells twice as large (0.69 mm^3^ in May and 0.36 mm^3^ in March in the control and warm stations, respectively). The minimum volume is also significantly higher (0.12 and 0.08 mm^3^, respectively, both during August). The calcite-to-shell volume ratio remains similar in both locations (Kruskal–Wallis *χ* ^2^ = 0.020038, d.f. = 1, *p*‐value = 0.8874) despite the reduced size in the warm station (figure 3) indicating that calcification is reducing proportionally to growth.

**Figure 4.**
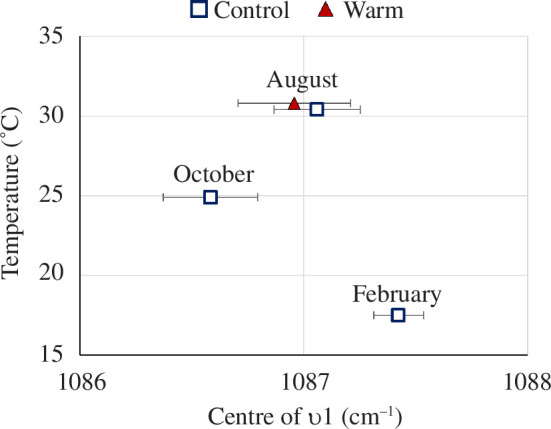
Centre position of the υ 1 Raman mode as a function of the average temperature at month of collection measured from the last chamber of shells from the control (empty squares) and warm (red full triangle) stations representing the full yearly temperature range. Error bars represent the standard error in the mean.

The position of the ν1 Raman mode in calcite is positively correlated to Mg concentration and hence predicted to increase with temperature [44]. In our results, the position of the ν1 mode is clustered by the time of sample collection rather than correlating to average temperature. Specifically, the position of the ν1 mode was lowest in the specimen collected in the control station during October (25°C), followed by the specimens collected during August (30°C and 31°C in the control and warm stations, respectively), and the highest in specimens collected in the control station during February (17°C) (figure 4). This lack of a positive, linear relationship between temperature and ν1 Raman mode position indicates that the calcite measured was not formed at the time of collection as it does not correlate to the temperature at that time. This mismatch is further strengthened by the significant differences observed between samples collected in February and in October (Kruskal–Wallis *χ* ^2^ = 9.3556, d.f. = 3, *p*‐value < 0.05, *p*-value for cold versus optimal < 0.01, electronic supplementary material, table S3) indicating these were precipitated under the most distinct conditions.

**Figure 5.**
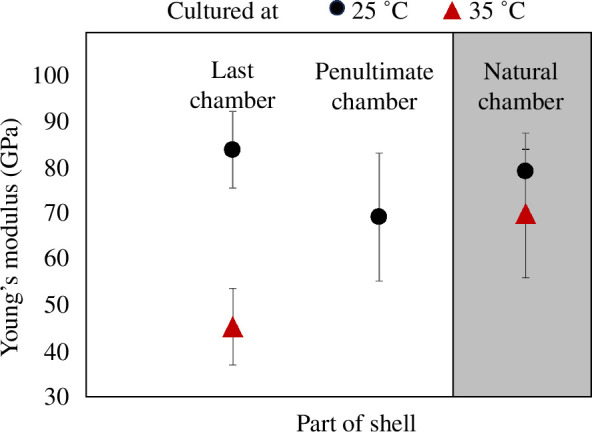
Young’s modulus of specimens grown in culture from the 25°C (black circle) and 35°C (red triangle) laboratory treatments. The last and penultimate chambers are precipitated in the experiment at the recorded temperature whereas the ‘natural chamber’ was precipitated before collection in the field where temperatures during growth are less well constrained (grey background). Error bars represent the standard error in the mean.

To be able to test the sensitivity of the FEA model for environmentally driven changes in the elasticity of the shells, we performed AFM measurements of Young’s modulus. As discussed in §2, we had to perform the measurements on a related genus, *O. ammonoides*, which has the same biomineralization mechanisms as *A. lobifera* [42] but a broader thermal range of calcification allowing it to grow in this temperature range. All 20 *O. ammonoides* specimens cultured at 25°C precipitated multiple new chambers while only three individuals calcified at 35°C producing only one new chamber each. Chambers that grew before culturing had Young’s moduli of 79 ± 8 GPa (*n* = 3 specimens later cultured at 25°C) and 70 ± 14 GPa (*n* = 3 specimens later cultured at 35°C) indicating the natural variability at the same conditions. Chambers precipitated at 25°C exhibited a similar Young’s modulus to the ‘natural chambers’ (created in the field before the experiment) of 69 ± 14 GPa (*n* = 3) in the penultimate chamber and 84 ± 8 GPa (*n* = 3) in the final chamber. A much lower Young’s modulus of 46 ± 9 GPa (*n* = 3) was found in the last chamber of specimens which grew under stress at temperatures of 35°C (figure 5).

### 3.2. Structural integrity and stress distribution

**Figure 6.**
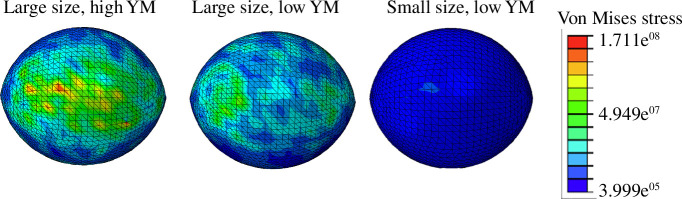
Outputs of FEA models showing the von Mises stress on shells under loads of 20 kPa with current and future shell parameters. The models represent current size based on average diameter and volume of shells from the control stations, and future size is reduced by 56% as indicated by shells from the warm stations. Young’s moduli used are 84 to 46 GPa (see §3).

To test the implications of the changes in morphology and material properties we generated a set of models reflecting the observed changes in size (table 1) and elasticity (Young’s modulus). Given that the largest morphological change between the stations was size, we tested its effect on the three generic models representing LBF morphology: a two-dimensional non-convex model containing the internal structure of the specimen, an external model reflecting the shape but without the internal structure and a three-dimensional biconvex external model (figure 2). Both non-convex models have similar von Mises stress values irrespective of changes in size between the stations, and in external stress loads (table 2). The convex model, in contrast, which is closer to the real geometry, shows higher average maximum von Mises stress values in the larger control specimen compared with the warm station model. Thus, the only parameter that significantly affects stress on the shells is the size of the convex shape. The reduction of the Young’s modulus from 84 to 46 GPa has no significant effect on the average stress levels of the external convex model (table 3). The only impact of the change in Young’s modulus is a change in stress distribution, which is more locally focused on the point of the highest curvature in the model with high elasticity (figure 6). The combined effect of size and Young’s modulus reduces the von Mises stress by approximately 8%.

**Table 2.** Von Mises stress values from the FE models, testing the effect of changes in size for each of the model types. The small model represents the change in size in the warm station and the large model represents foraminifera found at the control station.

	20 000 Pa				30 000 Pa			
model	control station (large model)	s.e.	warm station (small model)	s.e.	control station (large model)	s.e.	warm station (small model	s.e.
external	6.00E + 07	5.99E + 07	6.59E + 07	6.59E + 07	8.75E + 07	8.75E + 07	8.38E + 07	8.38E + 07
internal	4.72E + 06	4.69E + 06	6.40E + 06	6.39E + 06	7.02E + 06	6.98E + 06	9.72E + 06	9.70E + 06
convex	1.04E + 08	4.70E + 06	7.86E + 06	6.38E + 06	1.56E + 08	6.97E + 06	1.19E + 07	9.68E + 06

**Table 3.** Von Mises stress values from the finite element convex models testing the effect of changes in size, Young’s modulus and both combined, as expected in a future warmer world.

	avg.	s.e.
control station size, optimal temperature Young’s modulus	1.0E + 08	6.0E + 07
control station size, stress temperature Young’s modulus	7.3E + 07	4.2E + 07
warm station size, stress temperature Young’s modulus	7.9E + 06	4.6E + 06

## 4. Discussion

Our results show clear changes in foraminiferal growth in response to stress, impacting not only their final size but also the material properties of their shells. The potential impacts on the function of the foraminiferal shell both for the specimen but also for the ecosystem via carbonate production are explored here.

### 4.1. Impacts of warming on growth

The thermally disturbed area provides a test case focusing on local species which had time to acclimatize and adapt over decades and generations, making it a reliable test site for warming impacts while dampening the potential short-term reactions in culture [45]. Previous work at this location has shown that even the most thermally resilient foraminifera species, *Pararotalia calcariformata* and *Lachlanella* sp. reduce calcification, creating thinner or smaller shells [4]. In contrast, the species studied here, *A. lobifera*, is at the limit of its thermal tolerance [37] and therefore under threat from extirpation. Akin to other taxa, the most prominent observed change in morphology is a reduction of shell size, probably linked to changes in reproduction and a shorter life cycle at the warmer station allowing less time for growth. Shell thickness is not altered as the amount of calcite per volume stays stable. This is important as the wall thickness modifies the amount of light transferred to the symbionts within the shells and hence limits habitat depth with increasing thickness [16].

### 4.2. Impacts on metabolism and reproduction

Dwarfism in response to warming has been documented in many taxa and is consistent with the metabolic theory of ecology suggesting that warming increases metabolic rates, which, if they cannot be satisfied by food intake, impact biological processes including growth rates [46]. Higher metabolic rates at warmer temperatures may speed up growth, if sufficient energy is available, but can concurrently shorten an organism’s lifespan, as the size needed for reproduction is reached earlier, indicating a trade-off between growth and longevity, which impacts abundances and carbonate production [47]. Both planktic and benthic foraminifera show a general trend towards smaller shell sizes in response to warming or stress [4,38,48–51]. This strategy may allow reallocation of energy from routine functions that are not necessary for survival, to more fundamental metabolic processes [52–54].

### 4.3. Impacts on material properties in the field and in experiments

Changes in metabolic activity may impair the calcification process, leading to thinner shells [55], which intuitively are considered more fragile [56]. While it is established that size and thickness are negatively affected by warming [4,17,18,38,48,57], other parameters affecting robustness are less well investigated, such as material properties. In carbonates, changes to the crystal structure are reflected in their Raman spectra. Specifically, the position of the vibrational modes is positively correlated with Mg content and thus our winter specimens should have ν1 Raman modes with the lowest wavenumbers. Against expectations, our results show higher wavenumbers for shells collected in winter compared with all other thermal conditions suggesting they have higher Mg content and indicating calcification under higher temperatures. This demonstrates one of the difficulties of a field study: specimens collected at specific times did not precipitate new calcite under the specific conditions at that time, and not even during the month of collection (as shown also in Titelboim *et al*. [41] for other species). Hence, the high wavenumbers of the winter specimens indicate that *A. lobifera*, like other species in the Eastern Mediterranean [41], is not calcifying in winter and was in fact created during the previous summer. This interpretation is supported by the upper (32°C) and lower (15°C) calcification thresholds of this species documented in laboratory experiments [35,36].

An increase in the position of the ν1 Raman mode with temperature caused by increasing Mg content with warming has been shown to produce higher elastic modulus and hardness values in sea urchins [20]. Our AFM analysis provides the first quantification of the change in the elasticity of LBF shells under thermal stress, indicating a reduction of approximately 50%. This value is probably an underestimate as we had to choose a species (*O. ammonoides*), which is exceptionally able to calcify new chambers even at 35°C while other taxa stop growing before this temperature is reached. The similarity of the crystallographic changes between the two examined species (see §3) suggests a correlation between ν1 Raman mode position and Young’s modulus, which we were only able to establish on *O. ammonoides*. We anticipated that such a drastic reduction in calcium carbonate shell elasticity could make these organisms more vulnerable to predation and environmental stressors, thereby affecting their survival and potentially impacting the wider ecosystem services they provide.

### 4.4. Implications for structural integrity

To investigate the structural implications of observed changes in shell morphology and elasticity we used FEA. In other calcifiers, environmental stress reduced structural integrity via altering the growth geometry. For example, coralline algae create larger cells with thinner cell walls when exposed to high CO_2_ [10], while corals increase their skeletal porosity and reduce their bulk density and stiffness [58]. In both cases, structural integrity is impaired and damage susceptibility is increased [27,58]. Our models show that smaller biconvex oval shells, representing the warmer conditions, experience significantly lower stress under both low and high external loads indicating they will be more resilient to external stressors under future warming. We did not detect any changes to the internal structure of the foraminifers in the CT scans and therefore did not alter them in the models. Morphological plasticity is indeed documented in LBF as potentially beneficial: for example, more streamlined forms can burrow deeper into the sediment [59]. Further, our models show that the reduction of Young’s modulus, even by 50%, does not affect the shell's structural integrity. It is important to note that our experiment, like most FEA, does not consider the role of organics between calcite layers or around individual crystals which could play a role in enhancing hardness [60]. It has been shown in taxa with very different calcification processes and mineralogy that they increase skeletal organic content under conditions of ocean acidification [61–63] though this does not appear to impact hardness significantly [64]. Moreover, as foraminifera have very low organic content in their shells, ranging between 0.04% and 0.1% [65,66], we assume that this is not likely to have a significant effect. Furthermore, the 20 measurements done on seven different locations on each chamber wall would have most likely revealed anomalies in the elastic modulus caused by local differences in organics.

## 5. Conclusion

The unique case of increased structural integrity as a result of induced thermal stress has not been described before. Such an adaptation might explain the success of LBF in past environmental crises by giving them an advantage when handling external stressors and explaining ecological successions during warming [67,68]. Our results show that *A. lobifera* has acclimatized to thermal stress over multiple generations by reducing its size by 50% while maintaining a constant calcite-to-volume ratio. This is probably due to changes to its reproductive cycle, leading to shorter growth phases. In response to heat stress, the shell’s crystal structure changes with elasticity reduced by up to approximately 50%. FEA analysis demonstrated that this reduction of the Young’s modulus does not cause a significant change to the structural integrity of the shell. In contrast, the smaller size significantly strengthens the shell and its ability to protect the organism. Size reduction is prevalent in many foraminifera as a response to environmental stress. This observation implies that as warming progresses foraminifera will produce shells that are better adapted to endure enhanced physical stress, providing them with an advantage in a warmer world while at the same time reducing their reproductive success and abundance.

## Data Availability

All data underlying this article is available as a supplementary file [70]. The CT scans and FE models are available at the University of Bristol data repository, data.bris [69].
